# Effects of Habenular Stimulation Frequencies on Obstructive Sleep Apnea Induced by Stimulation of Insular Cortex

**DOI:** 10.1155/2018/9060678

**Published:** 2018-03-26

**Authors:** Jing Kang, Ming-Xian Li

**Affiliations:** ^1^The First Affiliated Hospital of Jilin University, Changchun 130021, China; ^2^Jilin Medical University, Jilin 132013, China

## Abstract

**Objective:**

To investigate the effects of high-frequency stimulation of the habenula (Hb) on obstructive sleep apnea (OSA) induced by stimulation of the insular cortex *Method*. After OSA was induced by stimulating the insular cortex (Ic) with concentric stimulating electrodes at 100 Hz in rats, the Hb was stimulated at different frequencies (50 Hz, 120 Hz, 130 Hz, and 280 Hz). The changes of apnea events and electromyography (EMG) of the genioglossus were compared before and after stimulation of the Hb.

**Results:**

With stimulation of the Ic at 100 Hz, apnea events were successfully induced with disappearance of EMG of the genioglossus. After stimulation of the Hb at 130 Hz, apnea events disappeared with significantly increased genioglossal EMG. However, such a change failed to be found at the stimulation frequencies of 50 Hz, 120 Hz, and 280 Hz.

**Conclusion:**

Stimulation of the Hb at the frequency of 130 Hz could effectively inhibit OSA events induced by stimulation of the Ic.

## 1. Introduction

Abnormal sleep position, alcohol consumption, drug intake, and obesity can induce obstructive sleep apnea (OSA) [1–3]. Such a complex and highly individualized disease has been associated with multiple cardiovascular diseases. OSA induces chronic hypoxia and hypoventilation that may cause pulmonary hypertension and pulmonary heart disease [4–7]. The predominant pathological causes of OSA include contractility of upper airway muscles, abnormal airway anatomical features, loop gain, and arousal threshold [8]. Since the genioglossus tongue muscle contraction stabilizes and enlarges the portion of the upper airway which is vulnerable to collapse, relaxation of the genioglossus has been implicated in OSA.

According to electroencephalography patterns, which can be used to confirm different stages of sleep, there is a higher likelihood for muscles around the upper airway to relax during deep sleep, thus increasing the possibility of OSA to occur [9]. Previous studies also demonstrated that the electromyography (EMG) activity of the genioglossus is reduced during deep sleep [9]. Subsequently, systolic function decreases with muscle relaxation, which might cause the pharyngeal wall to collapse [10]. Hence, stenosis or closure of the upper airway is usually associated with reduced ventilation or OSA [11–13]. Although recent advancement in neurophysiology has been made in understanding the role of neuronal activity in respiratory movement regulation [13], the pathways regulating the motor control of respiration is not fully explored, especially those regulating the genioglossus contraction, and could be involved in OSA pathogenesis.

Respiratory movement originates from the brainstem and could be regulated by chemoreceptors and mechanoreceptors [12]. In addition, the respiratory movement can also be influenced by signals from the upper portion of the pons and the cerebral cortex [6]. Functional magnetic resonance imaging (fMRI) and animal studies have implicated that the insula cortex plays a major role in the provoking of OSA [7, 8]. In addition, the insular cortex could cause cardiovascular system dysfunction, which is consistent in OSA patients [9]. On the other hand, previous studies have shown that the Hb can mediate the neuronal signaling from the insula cortex. Inhibition of the Hb was able to abrogate OSA induced by electrical stimulation of the insula cortex [10]. However, it is unclear whether the amplitude of the frequency used to stimulate the Hb is critical to achieve the maximum response in OSA. In this study, we investigated the effect of different Hb stimulation frequencies on obstructive sleep apnea. The breathing pattern as well as the EMG activities of the genioglossus was recorded before and after OSA was induced.

## 2. Materials and Methods

### 2.1. Animal Preparation

This study was carried out in accordance with the Guide for the Care and Use of Laboratory Animals of the National Institutes of Health. The protocol was approved by the Animal Care and Use Committee of the Jilin University (Permit number: SCXK, Kyrgyzstan, 2007-0003, and SYXK, Kyrgyzstan, 2007-0011). Wistar rats (200–280 g, 6–8 weeks old, *N*=92) were obtained from the Jilin University Norman Bethune Medical Department Laboratory Animal Center. The animals were housed under a 12/12-hour light/dark cycle in a temperature-controlled room (maintained at 22 ± 2°C) with ad libitum access to food and water [11]. All surgical and animal handling procedures were performed in accordance with Guidance Suggestions for the Care and Use of Laboratory Animals issued by the Ministry of Science and Technology of the People's Republic of China. The animals were randomly divided into three groups, the OSA group (*n*=24), Hb group (*n*=48), and control group (*n*=20), using SPSS software. For the OSA group, the insula cortex was stimulated to induce OSA. For the Hb group, the insula cortex and Hb were stimulated with four different frequencies (*n*=12 for each frequency). For the control group, the surrounding tissue of either the insula cortex (*n*=10) or the Hb (*n*=10) was stimulated. The breathing pattern and the EMG activities of the genioglossus and diaphragm were recorded before and after stimulation.

All experimental procedures were performed under anesthesia via intraperitoneal injection of 20% urethane (6 mL/kg), and all efforts were made to minimize suffering. During the surgery, the animals were breathing steadily and maintained in a state of light anesthesia (sensitive corneal reflex and forceps pinch produced withdrawal but no sustained response and no voluntary activities) [14] throughout the experimental procedure. Anesthesia was supplemented if needed during the experiment. The animals were maintained at 36°C rectal temperature with a heating pad. During the experiment, the animals were fixed under the stereotaxic instruments (Takahashi Company, Japan) with blunt ear bars, and a small craniotomy was performed. For the OSA group, an electrode was implanted into the insula cortex (anterior/posterior: 1.0 mm; medial/lateral: 5.4-5.5 mm; and dorsal/ventral: 4.2–4.4 mm), according to the rat brain atlas [15]. Whereas in the Hb group, electrodes were implanted into the insula cortex (Ic) and Hb (anterior/posterior: −3.2 mm; medial/lateral: 0.3–0.6 mm; and dorsal/ventral: 3.8–4.2 mm). The control group received electrical stimulation with the same parameters, but the electrode locations were at four points (top, bottom, left, and right) within a 1 mm^2^ range around the Ic. The rats were fixed in a ventral decubitus position and breathed spontaneously during the experiments.

### 2.2. Electrical Stimulation and Recording Protocols

**Figure 1:**
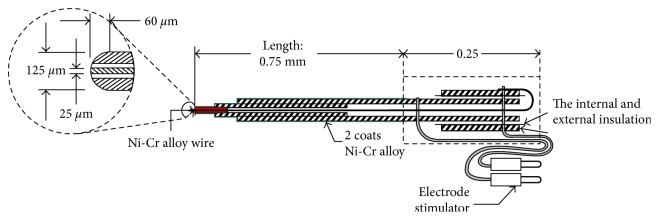
Schematic of stimulation electrode. The experimental stimulation electrode is a Ni-Cr alloy wire concentric exciter electrode of Japanese Narishige Company.

Electrical stimulation device (Nihon Kohden, Japan) and concentric stimulating electrodes (Nihon Kohden, Japan; outer diameter of 2.0 mm and impedance of 10–15 MΩ; Figure 1) were used for stimulation. OSA was induced by stimulating the Ic at 100 Hz, 0.4 mA, and 1 ms interval for 9 s [16]. Signs of apnea and respiratory distresses were used as indicators of successful induction during the experiment. Histology analysis of induction site was also performed. No signs of apnea and respiratory distress were observed in the control group. In the Hb group, the Hb was stimulated continuously for at least 30 min at 50 Hz, 120 Hz, 130 Hz, or 280 Hz before and after the insula cortex was stimulated.

The breathing pattern and EMG were recorded using a data logger (BL-420S, Chengdu Taimeng Technology Co., Ltd.). A respiratory flow transducer and a tension transducer were utilized to monitor the respiratory flow via endotracheal intubation and respiratory rates, respectively. The lead electrode was inserted to the genioglossus and diaphragm to record the EMG signals in the genioglossus and diaphragm, respectively.

### 2.3. Data Analysis

The electromyography (EMG) of the genioglossus and diaphragm, as well as the respiratory amplitude and respiratory rate, was compared before and after the stimulation of the Hb. SPSS 19.0 was used for all statistical analysis. Data were presented as mean ± standard deviation (*X* ± *S*). *P* < 0.05 was considered as statistically significant.

## 3. Results

**Figure 2:**
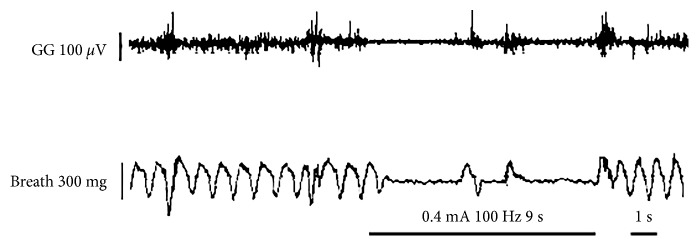
Typical changes in respiration and genioglossus EMG patterns before, during, and after stimulation in the IC with 100 Hz.

**Figure 3:**
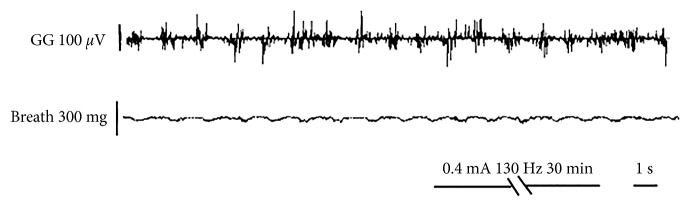
Typical respiration and genioglossus EMG patterns after 130 Hz stimulation of the Hb in the OSA-induced animal.

**Figure 4:**
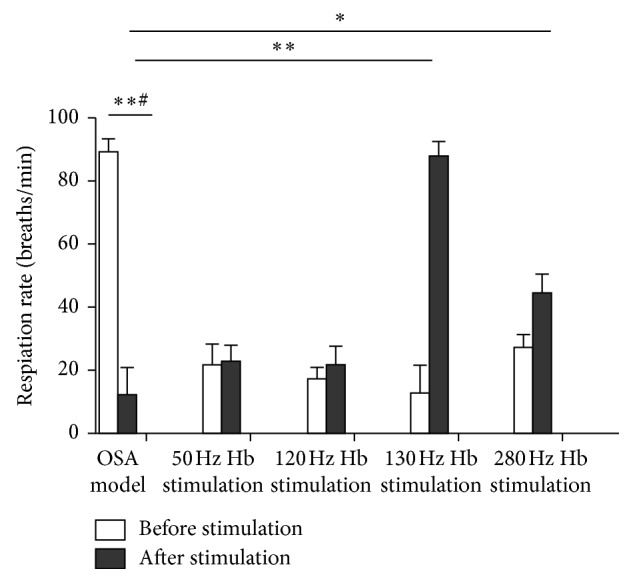
Respiration rate changes at different stimulation frequencies of the Hb in OSA-induced animal models. ∗∗# denotes *P* < 0.001 before and after stimulation in the insula cortex, while ∗∗ and ∗ denote *P* < 0.001 and *P* < 0.05, respectively, between the OSA model and different stimulation frequencies in the habenula.

Concentric stimulation of the Ic was performed in 12 OSA rats. During stimulation, weakened genioglossus contraction, apnea, interruption of airflow, and reduction of the respiratory rate were observed in OSA rats. Whereas, after stimulation, signs including intermittent apnea, a significant reduction of airflow (from 89.08 ± 4.21 breaths/min to 12.19 ± 8.49 breaths/min), transbreathing, reduction in the respiratory rate, deep breathing after stimulation, airflow return to baseline in few seconds, and significant reduction of genioglossus discharge were observed (Figure 2). Subsequently, the Hb was stimulated continuously for 30 min at 50 Hz, 120 Hz, 130 Hz, and 280 Hz in the same rat. Simultaneous concentric stimulation of the Ic was performed using the same parameters, and the breath and EMG activities of the genioglossus were monitored. As shown in Figure 3, the respiration rate and amplitude as well as EMG patterns of the genioglossus were returned within normal ranges after 130 Hz stimulation. Notably, the respiration rate changes at different stimulation frequencies of the Hb in OSA-induced animal models (Figure 4).

## 4. Discussion

The genioglossus has been commonly considered as the pathophysiology of OSA. Horner et al. reported that the genioglossus contractile function was significantly decreased during sleep [17]. Our results showed a reduction in genioglossus contraction in OSA rats, which is consistent with a previous study showing that genioglossus activity significantly reduced during sleep in OSA patients with subsequent weakening in contraction resulting in muscle relaxation in the pharyngeal wall [18]. As a consequence, a reduction in the respiratory rate was observed (Figure 2), which may be attributed to the subsequent collapse of the upper airway, thus reducing ventilation [18]. It should be noted that, despite the application of excitatory neurotransmitter serotonin (5-hydroxytryptamine, 5-HT), sleep could not prevent genioglossus diastole [19]. Thus, the main reason for the occurrence of OSA is associated with respiratory disorders, suggesting that the genioglossus could play an important role in OSA. The genioglossus electrical activity weakens during sleep, causing the muscles to relax and a reduction in ventilation. Consequently, chronic intermittent hypoxia or hypercapnia (chronic intermittent hypopnea or hypercapnia, CIHH) occurs, or even acidosis.

Our results showed that 100 Hz concentric stimulation of the Ic could induce OSA in rats. The Ic, a region of the limbic system, can change the genioglossus tension, as well as the breathing depth and rate. Many neurotransmitters regulating the limbic system are associated with many diseases [20–23]. Signals propagating from the Ic to the Hb could reduce the release of 5-HT in the raphe nuclei. Subsequently, the activity of hypoglossal nucleus would decrease, resulting in the reduction of genioglossus contractile function. Consequently, genioglossus relaxation occurs, which can cause airway to collapse, and thus OSA. It should be mentioned that the Ic is involved in regulating OSA and has been related to respiratory motion adjustments [11]. The activities in the Ic increased during OSA, and inhibition could occur in the raphe nucleus that reduces the release of 5-HT. Subsequently, the activity of the hypoglossal nucleus and the hypoglossal nerve excitability are reduced. Thus, the genioglossus contraction is weakened, and the upper airway will be more vulnerable to collapse causing OSA.

When the Hb was stimulated continuously for 30 min at 130 Hz, the EMG patterns of the genioglossus returned within normal ranges. These results indicate that such a stimulation can be used to treat OSA. The Hb plays an important role in OSA, since the Hb mediates signals from the Ic to the downstream. Consequently, 130 Hz stimulation may block the Hb, resulting in genioglossus contraction, and thus, the upper airway may open and ultimately improve ventilation. These results indicate that inhibition of the Hb can block downstream signal propagation from the Ic, thereby avoiding the formation of OSA. Our results reveal the relationship between Hb, OSA, and Ic and may also provide a new therapeutic strategy in OSA.

## 5. Conclusion

Electrical stimulation of 130 Hz could inhibit Hb, which reduces downstream signal propagation initiated by the Ic. This, in turn, will cause the genioglossus contractile function to recover, avoiding the formation of obstructive sleep apnea.
